# Growth Hormone-Regulated mRNAs and miRNAs in Chicken Hepatocytes

**DOI:** 10.1371/journal.pone.0112896

**Published:** 2014-11-11

**Authors:** Xingguo Wang, Lei Yang, Huijuan Wang, Fang Shao, JianFeng Yu, Honglin Jiang, Yaoping Han, Daoqing Gong, Zhiliang Gu

**Affiliations:** 1 Department of Life Science and Technology, Changshu Institute of Technology, Changshu, P R China; 2 College of Animal Science and Technology, Yangzhou University, Yangzhou, P R China; 3 Department of Animal and Poultry Sciences, Virginia Polytechnic Institute and State University, Blacksburg, Virginia, United States of America; Laboratoire de Biologie du Développement de Villefranche-sur-Mer, France

## Abstract

Growth hormone (GH) is a key regulatory factor in animal growth, development and metabolism. Based on the expression level of the GH receptor, the chicken liver is a major target organ of GH, but the biological effects of GH on the chicken liver are not fully understood. In this work we identified mRNAs and miRNAs that are regulated by GH in primary hepatocytes from female chickens through RNA-seq, and analyzed the functional relevance of these mRNAs and miRNAs through GO enrichment analysis and miRNA target prediction. A total of 164 mRNAs were found to be differentially expressed between GH-treated and control chicken hepatocytes, of which 112 were up-regulated and 52 were down-regulated by GH. A total of 225 chicken miRNAs were identified by the RNA-Seq analysis. Among these miRNAs 16 were up-regulated and 1 miRNA was down-regulated by GH. The GH-regulated mRNAs were mainly involved in growth and metabolism. Most of the GH-upregulated or GH-downregulated miRNAs were predicted to target the GH-downregulated or GH-upregulated mRNAs, respectively, involved in lipid metabolism. This study reveals that GH regulates the expression of many mRNAs involved in metabolism in female chicken hepatocytes, which suggests that GH plays an important role in regulating liver metabolism in female chickens. The results of this study also support the hypothesis that GH regulates lipid metabolism in chicken liver in part by regulating the expression of miRNAs that target the mRNAs involved in lipid metabolism.

## Introduction

Growth hormone (GH) is a peptide hormone from the anterior pituitary gland [1], [2]. It has many biological effects at both the whole body and tissue levels [3]. GH regulates animal growth, development and metabolism [3], [4], [5], [6]. GH regulates the metabolism of not only protein but also that of lipid and carbohydrates [7]. GH initiates its function by binding to the GH receptor (GHR) [8], [9], [10]. Binding of GH to the GHR activates the receptor-associated tyrosine kinase JAK2 [11], and JAK2 then activates multiple proteins, including STAT1, STAT3, STAT5, MAPK, and PI3K [12]. These proteins in turn mediate GH-caused changes in gene expression or protein modification.

Liver is a key metabolic organ, and in chickens this is where most of the *de novo* synthesis of fatty acids occurs [13], [14]. microRNAs (miRNAs) are a class of small non-coding RNAs about 22 nucleotides in length, and regulate gene expression by interacting with the 3′ untranslated regions (UTRs) of target mRNAs [15]. miRNAs have been shown to play important roles in many biological processes including liver metabolism. For example, miR-122, abundantly expressed in liver, modulates protein metabolism in liver by targeting cationic amino acid transporter 1 [16]; it regulates the synthesis of fatty acids and cholesterol by repressing the expression of aldolase-A, 3-hydroxy-3-methylglutaryl-coenzyme A reductase, and AMP-activated protein kinase [17], [18]. miR-33 is another miRNA involved in liver metabolism: it regulates cholesterol efflux and high-density lipoprotein metabolism by targeting ATP-binding cassette, sub-family A (ABC1), member 1 and ATP-binding cassette, sub-family G (WHITE), member 1 [19], and it reduces fatty acid degradation by targeting multiple genes involved in fatty acid β-oxidation [20].

RNA sequencing (RNA-seq) is a novel gene expression profiling technology based on high-throughput DNA sequencing. The benefit of RNA-Seq over other large-scale gene expression profiling methods is its ability to measure mRNA expression in a single assay and, at the same time, reveal new genes and transcripts [21],[22],[23]. Similarly, RNA-seq can be also used to identify novel miRNAs and detect differentially expressed miRNAs between samples [24].

As in mammals [3], [7], [25], [26], GH has metabolic effects in chickens: GH regulates lipid metabolism in chicken adipose tissue [27] and chicken liver [28]. However, it is unclear whether GH has the same growth-stimulating effect in chickens as in mammals because exogenous GH treatment to chickens causes no growth responses and because plasma GH concentrations in chickens are in general not correlated with growth rates [29].

The mechanism by which GH regulates lipid metabolism in chicken liver is not clear. In this study, we determined the effects of GH on the expression levels of all mRNAs and miRNAs in primary hepatocytes from female chickens by RNA-seq. We analyzed the differentially expressed mRNAs or genes (DEG) and differentially expressed miRNAs (DEM) with multiple bioinformatics tools to correlate the DEG and the DEM to the physiological functions of GH. The main hypothesis to be tested in this study was that GH regulates liver metabolism in the chicken in part by regulating the expression of miRNAs that target mRNAs directly related to liver metabolism.

## Materials and Methods

### 1. Culture of primary chicken hepatocytes

All procedures involving animals were approved by Changshu Institute of Technology Institutional Animal Care and Use Committee and conformed to the Guide for the Care and Use of Laboratory Animals of Jiangsu Province. All efforts were made to minimize suffering. Primary chicken hepatocytes were isolated from 4 female, 4-week-old Arbor Acres commercial chickens which were fasted 12 hours (h) before being anaesthetized by intraperitoneal injection of sodium thiopenthal (50 mg/kg) and anticoagulated by intraperitoneal injection of heparin (1750 U/kg). Livers were isolated and the chickens were sacrificed by removal of the hearts. Hepatocytes were isolated from the livers as previously described [30]. Hepatocytes from individual chickens were cultured separately. Hepatocytes were plated in 24-well plates or 10-cm dishes at a density of 1.3×10^6^ cells/ml in Willam’s E medium (Gibco, Grand Island, NY) supplemented with 5% chicken serum, 100 U/ml penicillin-streptomycin, 10 µg/ml insulin and 30 mmol/L NaCl in a humidified incubator at 37°C with 5% CO_2_. Twenty-eight h later, hepatocytes were serum starved for 8 h, followed by 12 h of treatment with 500 ng/ml chicken GH (chGH) (Prospec, Ness-Ziona, Israel) or an equal volume of PBS. Cells were lysed and total RNA was isolated using TRIzol reagent (Invitrogen, Carlsbad, CA) following the manufacturer’s directions.

### 2. Real time RT-PCR

Concentrations and quality of RNA samples were determined by NanoDrop ND2000 spectrophotometry (Thermo Scientific, Wilmington, DE) and formaldehyde-agarose gel electrophoresis. Five hundred ng of total RNA was reverse-transcribed to cDNA in a total volume of 10 µl using Takara PrimeScript RT reagent kit (Takara, Dalian, China). The relative expression levels of genes were quantified using SYBR Premix Ex Taq (Takara) on an Applied Biosystems 7500 Thermocycler (Applied Biosystems) according to the manufacturer’s directions. In this analysis, GAPDH was used as an internal control. All reactions were run in duplicate. Data are means ± SEM of 4 independent cell culture experiments (i.e., 4 chickens) and analyzed by student’s t-test. The primers for real time RT-PCR were presented in Table S1.

### 3. mRNA sequencing

RNA samples for mRNA sequencing were prepared using Illumina TrueSeq RNA Sample Preparation Kit according to the manufacturer’s directions. The liver RNA samples from 4 chGH-treated or PBS-treated chickens were pooled in equal volumes after their concentrations were adjusted to 10 nM. Sequencing of the cDNA was performed by Personalbio (Shanghai, China) using the Illumina Miseq system.

### 4. mRNA analysis

The reads obtained from sequencing were mapped to the chicken genome (WASHUC2.69) in Ensembl using Bowtie/Tophat (2.0.5) (http://tophat.cbcb.umd.edu), and the reads of each gene were normalized using reads per kilo bases per million reads (RPKM). The significance was determined by normalizing the raw reads and calculating the P-value using DESeq (http://bioconductor.org/packages/release/bioc/html/DESeq.html). Genes with fold change (RPKM (chGH/PBS)) >1.5 or <2/3 and *P*-value <0.05 were identified as DEG.

Gene Ontology (GO) enrichment analysis was performed using GOslim (http://www.geneontology.org/page/go-slim-and-subset-guide). The data were presented as −log (P-value). P-value = 1−
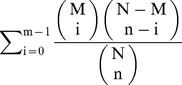
, where N is the total gene number in genome, n is the number of all DEG, M is the number of genes in genome that are involved in certain GO, and m is the number of DEG that are involved in certain GO. The signaling pathways were analyzed using the KEGG database.

### 5. Small RNA sequencing

The RNA samples used for small RNA sequencing were the same as for mRNA sequencing. Small RNA libraries for small RNA sequencing were constructed according to the Miseq small RNA sequencing protocol. Briefly, total RNA was ligated to the 3′ adapter. Then it was ligated to the 5′ adapter. The ligated RNA was reverse-transcribed to cDNA. The cDNA which had a 3′ adapter and a 5′ adapter was amplified by PCR. The PCR products with appropriate length were extracted from polyacrylamide gels to construct the small RNA libraries. The libraries of four samples were pooled in equal volumes after first being normalized to 10 nM. Sequencing of the small RNA libraries was performed by Personalbio using the Illumina Miseq system.

### 6. miRNA analysis

The raw reads from small RNA sequencing were treated by trimming the adapters and removing the low-quality sequences to obtain the clean reads. The clean reads with 15–30 nt were analyzed by counting and grouping identical sequences as unique reads. The unique reads were mapped to the chicken genome using Bowtie and BLAST searched against the ncRNA database Rfam (10.1) assess the quality of sequences and obtain ncRNA annotation. The sequence reads were first searched against the chicken miRNA database in miRBase (20.0) to identify known chicken miRNAs, then against the miRNA databases of other species in miRBase to identify chicken miRNAs homologous to known miRNAs in other species, and finally against the chicken genome to obtain their ∼80 nt genomic sequences flanking the 5′ or 3′ end and analyzed using the program mireap (http://sourceforge.net/projects/mireap/) to predict potentially novel chicken miRNAs and their precursors according to the miRNA biogenesis principle [31].

The reads of miRNAs from control (PBS treated) samples and GH treated samples were normalized using reads per million (RPM). RPM = Actual miRNA count/Total count of clean reads*1000000. When the RPM of a certain miRNA in one type of samples was zero, it was revised to 0.01, and if the RPM of a certain miRNA in both types of samples was less than 1, the miRNA would not be used in further DEM analysis. Fold change and P-value were calculated from the RPM. Fold change = RPM (chGH)/RPM (PBS). P-value formula: 
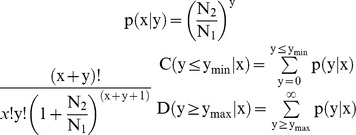
. The x and y represent normalized expression level, and the N1 and N2 represent total count of clean reads of a given miRNA in small RNA libraries of chGH and PBS groups, respectively. The miRNAs with fold change (RPM (chGH/PBS)) >1.5 or <2/3 and P-value <0.05 were identified as DEM [32].

Target genes of identified known chicken DEM were predicted in the DEG using miRanda with the TargetScan principle [30]. In brief, the target mRNAs of GH up-regulated miRNAs were predicted in GH down-regulated mRNAs; the target mRNAs of GH down-regulated miRNAs were predicted in the GH up-regulated mRNAs. Then GO enrichment analysis of GH-regulated mRNAs targeted by GH-regulated miRNAs was performed using the GOslim program.

## Results

### 1. mRNA expression profiling by RNA-seq

The RNA-seq generated 16,474,842 and 10,662,250 paired end reads from chGH-treated group and PBS-treated group, respectively, with an average length of 151 bp. After filtering with Q30, 13,638,494 and 7,473,018 useful reads were obtained from the treated group and control group, respectively. We mapped the useful reads to the chicken genome using bowite/tophat software, and found that 75.64% and 78.23% of reads in the treated group and control group mapped to the chicken genome, respectively. Of them, 82.59% in the treated group and 82.38% in the control group can be mapped to genes (Table 1). A total of 16,736 genes were identified.

**Table 1:** An overview of mRNA sequencing results.

Sample	Raw Reads	Clean Reads	Reads mapped to chicken genome	Reads mapped to chicken genes
chGH	16,474,842	13,638,494	10,669,529	8,812,163
PBS	10,662,250	7,473,018	5,652,712	4,656,574

Among the identified 16,736 genes, 164 genes with fold change >1.5 and *p*-value <0.05 were identified as DEG, of which 112 were up-regulated and 52 were down-regulated by chGH treatment (Table S2). Table 2 shows the top 10 up-regulated DEG and the top 10 down-regulated DEG.

**Table 2:** Top 10 GH up-regulated and top 10 GH down-regulated mRNA-encoding genes.

Name	PBS (RPKM)	chGH (RPKM)	fold change (RPKM (chGH/PBS))	P-value
**up-regulated genes**				
RPS28	0.52	21.63	40.74	0.0005
TTLL3	1.09	23.57	21.41	0.028
CISH	1.97	27.94	13.99	1.38E-10
SCN4A	1.87	20.79	10.95	5.29E-21
BLB1	0.74	7.13	9.53	0.009
TGM2	0.20	1.78	8.88	0.037
SS1R	0.68	5.33	8.75	0.015
SERPINA4	8.86	78.26	8.70	1.06E-18
GGA.45581	35.97	278.79	7.64	9.82E-31
CCK	3.23	24.44	7.46	5.05E-05
**down-regulated genes**				
AFP	38.74	0.10	0.0025	4.76E-40
RGS6	1.92	0.09	0.047	0.014
STAR	27.75	5.01	0.18	1.28E-08
CCKAR	5.57	1.01	0.18	0.013
KIAA0408	3.38	0.63	0.19	0.020
BCL6	19.86	4.26	0.21	8.27E-08
PDE10A	7.07	1.57	0.22	0.002
ENSGALG00000024377	26.06	6.18	0.23	0.044
NECAB1	7.12	1.74	0.24	0.010
TAGLN3	34.96	8.62	0.24	5.64E-06

To confirm the result of RNA-seq, we classified the genes into four groups: 1) fold change (RPKM (chGH/PBS)) >1.5 and P<0.05, 2) fold change (RPKM (chGH/PBS)) <2/3 and P<0.05, 3) fold change (RPKM (chGH/PBS))>1.5 and P>0.05, 4) fold change (RPKM (chGH/PBS)) <2/3 and P>0.05. And real-time RT-PCR was performed on a total of 39 genes that were randomly selected from four groups. Among them, 21 genes that were not differentially expressed between chGH and PBS based on RNA-seq were also found to be not differentially expressed by real-time RT-PCR, and 11 of 18 genes that were found to be differentially expressed by RNA-seq were also confirmed to be differentially expressed by real time RT-PCR (Table 3). Thus, our RNA-seq results were in general confirmed by real-time RT-PCR.

**Table 3:** Real time RT-PCR validation of gene expression levels revealed by RNA-seq.

Gene	Real-time RT-PCR		RNA-seq	
	Fold change^1^	P-value	Fold change^2^	P-value
**Group 1** 3				
FABP1	4.30	0.034	3.96	3.82E-07
FGFR3	2.37	3.93E-04	2.11	0.009
FURIN	1.71	0.012	2.30	2.82E-05
IRF8	2.68	0.005	2.50	1.69E-05
LPIN1	1.81	0.049	1.78	1.45E-04
MAPKAPK3	1.68	0.021	2.24	0.010
PHGDH	1.12	0.748	1.54	0.004
PKIG	1.47	0.094	1.67	0.046
THRSP	1.44	0.565	1.76	0.007
**Group 2** 4				
ABCG8	0.28	0.048	0.33	2.14E-04
ALDH1A3	0.70	0.087	0.66	0.009
BCL6	0.18	0.003	0.21	8.27E-08
LPIG	0.33	5.69E-04	0.34	2.67E-07
NECAB1	0.43	0.447	0.24	0.010
PDE10A	0.30	0.042	0.22	0.002
PPAP2B	0.61	0.075	0.52	0.010
RGS6	0.49	0.218	0.05	0.014
STAR	0.17	0.024	0.18	1.28E-08
**Group 3** 5				
FGF1	1.19	0.743	1.62	0.661
GPX7	1.15	0.760	3.66	0.552
MEF2A	0.90	0.665	6.79	0.461
NME1	0.90	0.800	7.83	0.384
PDGFB	0.78	0.350	1.72	0.694
PNPLA4	0.90	0.656	1.99	0.449
PRKAR2B	1.47	0.339	4.44	0.438
PTK2B	0.97	0.925	2.27	0.220
PTPRC	1.21	0.540	1.72	0.247
ROMO1	0.84	0.205	2.13	0.168
**Group 4** 6				
ACER2	1.30	0.163	0.46	0.437
ADPGK	1.02	0.759	0.56	0.371
ATF7	1.14	0.602	0.31	0.445
BIRC5	0.93	0.753	0.26	0.685
CDK2AP1	1.23	0.613	0.28	0.384
DUSP28	0.90	0.628	0.17	0.471
FTO	1.33	0.159	0.39	0.472
HGF	1.87	0.463	0.49	0.470
MAFK	0.83	0.074	0.50	0.144
NT5M	1.30	0.497	0.45	0.488
RUNX2	0.98	0.939	0.157	0.272

1 fold change by real-time RT-PCR is relative gene expression level (chGH/PBS).

2 fold change by RNA-seq is RPKM (chGH/PBS).

3 Group 1: fold change (RPKM (chGH/PBS)) >1.5 and P<0.05.

4 Group 2: fold change (RPKM (chGH/PBS)) <2/3 and P<0.05.

5 Group 3: fold change (RPKM (chGH/PBS)) >1.5 and P>0.05.

6 Group 4: fold change (RPKM (chGH/PBS)) <2/3 and P>0.05.

### 2. Functional analysis of differentially expressed mRNAs

**Figure 1:**
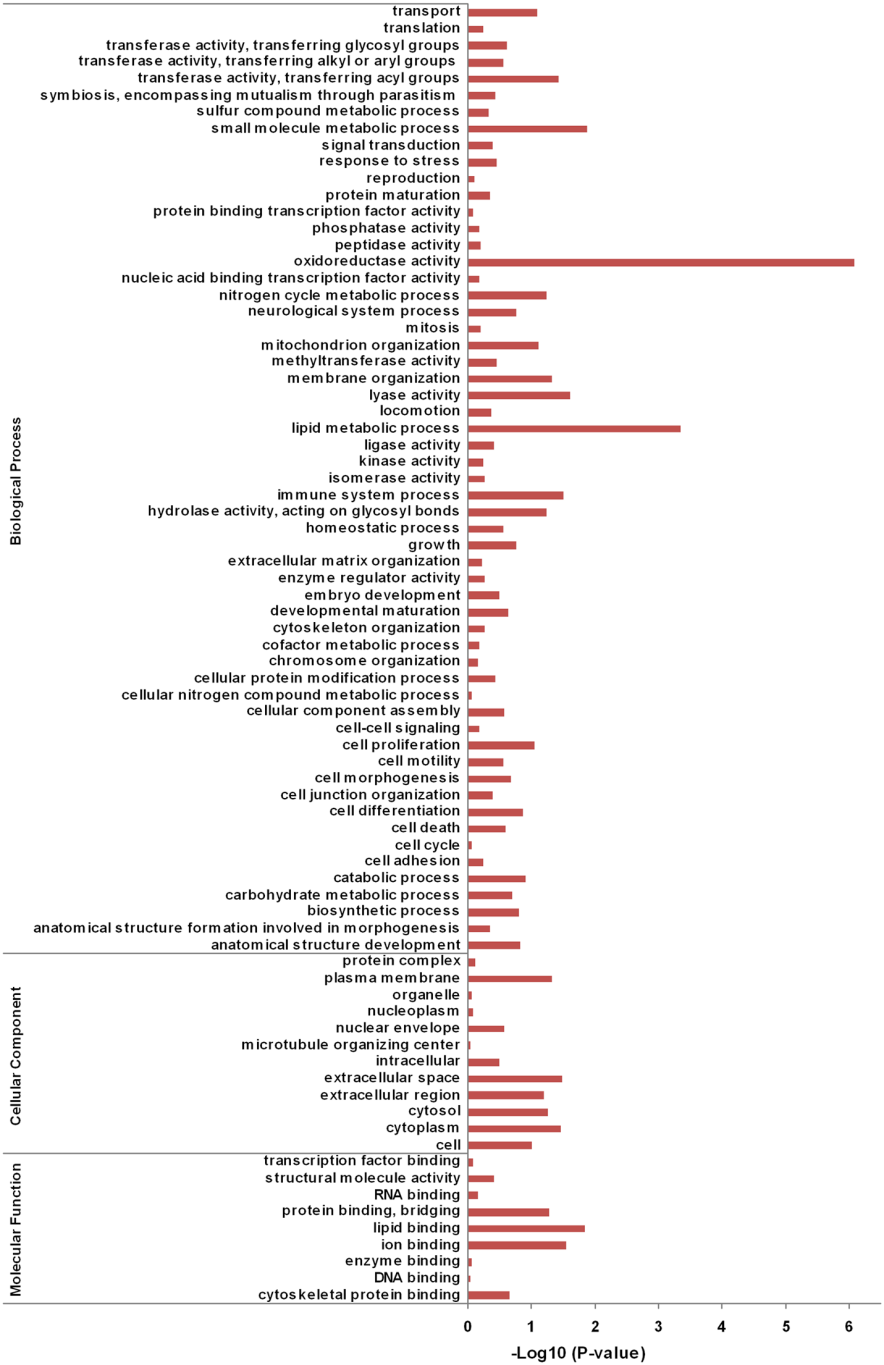
GO enrichment analysis of genes regulated by chGH in chicken hepatocytes. The GO terms are sorted by –Log10 of the enrichment P-value, which represents the enrichment significance of GO terms. The enrichment of GO terms is showed by comparing DEG with the whole genome from this figure.

To correlate the differentially expressed mRNAs with biological functions, we analyzed the functional bias of the DEG according to Gene Ontology enrichment. The analysis showed that the DEG included many genes involved in lipid binding, ion binding, transferase activity, oxidoreductase activity, immune system process, lyase activity, and lipid metabolic process. Other enriched GO terms included growth, cellular nitrogen compound metabolic process, carbohydrate metabolic process, and biosynthetic process (Figure 1). Most of the KEGG Orthology (KO) terms were related to lipid metabolism (Table S3). Among the GH up-regulated genes, three were involved in the pathways controlling lipid metabolism: *AKR1D1* was involved in primary bile acid biosynthesis and steroid hormone biosynthesis pathway, *FABP1* was involved in PPAR and fat digestion and absorption pathway, and *LPIN1* was involved in glycerolipid metabolism pathway. Among the GH down-regulated genes, only one was involved in the pathway related to lipid metabolism: *PPAP2B* was involved in glycerolipid metabolism and fat digestion and absorption pathway.

#### 2.1. Differentially expressed mRNAs involved in growth

We found that there were 6 GH-regulated genes involved in animal growth. Of them, *CISH*, *ULK2*, *IRF8*, and *FOXP2* were up-regulated by chGH, and *Bcl6* and *ROBO1* were down-regulated by chGH (Table 4). Both *Bcl6* and *ROBO1* are negative regulators of cell growth [33], [34]. This indicates that chGH may regulate chicken growth by both increasing the expression of genes that positively regulate growth and reducing the expression of genes that negatively regulate growth.

**Table 4:** List of growth-related mRNAs whose expression in chicken hepatocytes was regulated by chGH.

Name	PBS (RPKM)	chGH (RPKM)	fold change (RPKM (chGH/PBS))	P-value
BCL6	19.86	4.26	0.21	8.27E-08
CISH	1.97	27.94	13.99	1.38E-10
FOXP2	11.40	19.73	1.71	0.026
IRF8	25.75	65.31	2.50	1.69E-05
ROBO1	11.86	7.27	0.60	0.038
ULK2	23.90	70.81	2.92	2.97E-09

RPKM was denoted as a formula: 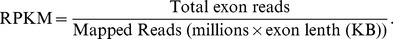

#### 2.2. Differentially expressed mRNAs related to metabolism

A total of 46 GH-regulated DEG were found to participate in protein, carbohydrate and lipid metabolism. Among them, 26 were related to protein metabolism as shown in Table 5, of which 20 were up-regulated and 6 were down-regulated by chGH, suggesting that GH plays an important role in protein metabolism in chicken hepatocytes. Further analysis showed that most of the 26 genes either promote protein synthesis or inhibit protein degradation, consistent with a previous study [7]. In addition to protein metabolism, 5 genes were related to carbohydrate metabolism, and all of them were up-regulated by chGH (Table 5). The specific functions of these genes include carbohydrate binding, transferase activity, and gluconeogenesis. As shown in Table 5, 15 DEG were related to lipid metabolism. Among these 15 genes, 8 showed increased expression after chGH treatment and all have oxidoreductase activity, suggesting that GH may promote lipid oxidation in chicken hepatocytes. Most of the 7 down-regulated genes were related to unsaturated fatty acid and long chain fatty acid biosynthesis, suggesting that GH may inhibit lipid synthesis in chicken hepatocytes.

**Table 5:** List of metabolism-related mRNAs whose expression in chicken hepatocytes was regulated by chGH.

Name	PBS (RPKM)	chGH (RPKM)	fold change (RPKM (chGH/PBS))	P-value
Protein metabolism				
AASS	35.18	53.89	1.51	0.029
ABCG5	15.51	7.35	0.47	0.018
ABCG8	20.88	6.94	0.33	2.14E-04
AKAP13	32.52	21.50	0.65	0.02311
BCL6	19.86	4.26	0.21	8.27E-08
CACNA1D	9.77	4.62	0.47	6.47E-04
CAMK1D	16.62	30.05	1.78	0.049
CEBPA	2.90	13.19	4.48	0.018
FGFR3	8.20	17.53	2.11	0.009
FOXP2	11.40	19.73	1.71	0.026
FZD5	22.61	46.43	2.02	0.021
F1NQS2	76.69	122.69	1.58	0.013
HAL	165.14	260.84	1.56	0.005
HistoneH4	12.98	36.83	2.79	0.0498
ICER	44.32	71.76	1.60	0.023
IRF8	25.75	65.31	2.50	1.69E-05
LPIN1	259.93	468.47	1.78	1.45E-04
MC5R	10.41	26.56	2.52	0.009
OXA1L	3.28	16.92	5.09	0.030
PHGDH	542.39	848.66	1.54	0.004
PKIG	163.59	277.14	1.67	0.046
PPAP2B	43.27	22.99	0.52	0.010
RAPGEF2	30.14	55.26	1.81	7.43E-04
RNL2	158.78	257.55	1.60	0.013
SLC7A2	19.42	30.91	1.57	0.048
UPP2	130.97	229.47	1.73	0.002
Carbohydrate metabolism				
CHIA	59.33	141.04	2.35	3.04E-06
E1BZP4	31.08	122.15	3.88	1.3E-10
MAN2A2	2.87	6.39	2.20	0.041
PCK2	51.79	102.40	1.95	0.003
ST6GAL1	33.42	54.46	1.61	0.018
Lipid metabolism				
ABHD5	23.45	47.53	2.00	8.61E-04
ACSL3	27.37	16.69	0.60	0.027
AKR1D1	0.85	4.83	5.57	0.043
ALDH1A3	148.38	99.67	0.66	0.009
ALDH8A1	108.87	174.87	1.58	0.008
CEBPA	2.90	13.19	4.48	0.018
ELOV6	196.41	117.72	0.59	0.005
ICER	44.32	71.76	1.60	0.023
LIPG	77.96	26.78	0.34	2.67E-07
LPIN1	259.93	468.47	1.78	1.45E-04
PLCD1	139.45	87.34	0.62	0.003
PPAP2B	43.27	22.99	0.52	0.010
RETSAT	18.49	37.63	2.01	0.024
STAR	27.75	5.01	0.18	1.28E-08
THRSP	74.53	133.21	1.76	0.007

#### 2.3. Differentially expressed mRNAs related to signaling pathways

We also identified which GH-regulated genes might be involved in intracellular signaling. The analysis indicated that 7 GH-regulated genes have DNA binding activity, of which 5 were up-regulated and 2 were down-regulated by chGH, and 24 GH-regulated genes were components of signal transduction pathways, of which 11 were up-regulated and 13 were down-regulated by chGH (Table 6). These pathways include the STAT and MAPK pathways, which are known to be activated by GH [35], [36] and pathways that are not known to be activated by GH.

**Table 6:** List of signal transduction-related mRNAs whose expression in chicken hepatocytes was regulated by chGH.

Name	PBS (RPKM)	chGH (RPKM)	fold change (RPKM (chGH/PBS))	P-value
Nucleic acid binding transcription factor				
BCL6	19.86	4.26	0.21	8.27E-08
CAMK1D	16.62	30.05	1.78	0.049
CEBPA	2.90	13.19	4.48	0.018
FOXP2	11.40	19.73	1.71	0.026017
ICER	44.32	71.76	1.60	0.023
IRF8	25.75	65.31	2.50	1.69E-05
PPAP2B	43.27	22.99	0.52	0.010
Signal transduction				
AKAP13	32.52	21.50	0.65	0.023
ALDH1A3	148.38	99.67	0.66	0.009
ARL5A	18.46	33.45	1.79	0.042
BCL6	19.86	4.26	0.21	8.27E-08
CACNA1D	9.77	4.62	0.47	6.47E-04
CBLB	5.26	2.37	0.44	0.046
CCKAR	5.57	1.01	0.18	0.013
CISH	1.97	27.94	13.99	1.38E-10
FGA	140.08	247.03	1.74	5.29E-04
FGFR3	8.20	17.53	2.11	0.009
FGG	111.03	176.06	1.56	0.009
FURIN	21.31	49.79	2.30	2.82E-05
FZD5	22.61	46.43	2.02	0.021
GPR1	49.72	24.24	0.48	0.005
SS1R	0.68	5.33	8.75	0.015
MAPKAPK3	7.28	16.57	2.24	0.010
MC5R	10.41	26.56	2.52	0.009
NCAM1	6.36	3.17	0.49	0.043
PDE10A	7.07	1.57	0.22	0.002
PLCD1	139.45	87.34	0.62	0.003
PPAP2B	43.27	22.99	0.52	0.010
RAPGEF2	30.14	55.26	1.81	7.43E-04
RGS6	1.92	0.09	0.047	0.014
SPTBN1	151.29	101.93	0.66	0.007

### 3. miRNA profiling by RNA-seq

Small RNA libraries of chGH-treated and PBS-treated hepatocytes were deep sequenced, generating 8,248,771 raw reads from PBS-treated hepatocytes and 5,232,471 raw reads from chGH-treated cells. Of these sequences, 5,240,376 and 2,144,128 were clean reads. A total of 3,124,826 clean reads and a total of 1,487,885 clean reads from PBS-treated cells and chGH-treated cells, respectively, were found to be between 15 and 30 nt in length, and these reads were considered as potential miRNAs.

**Figure 2:**
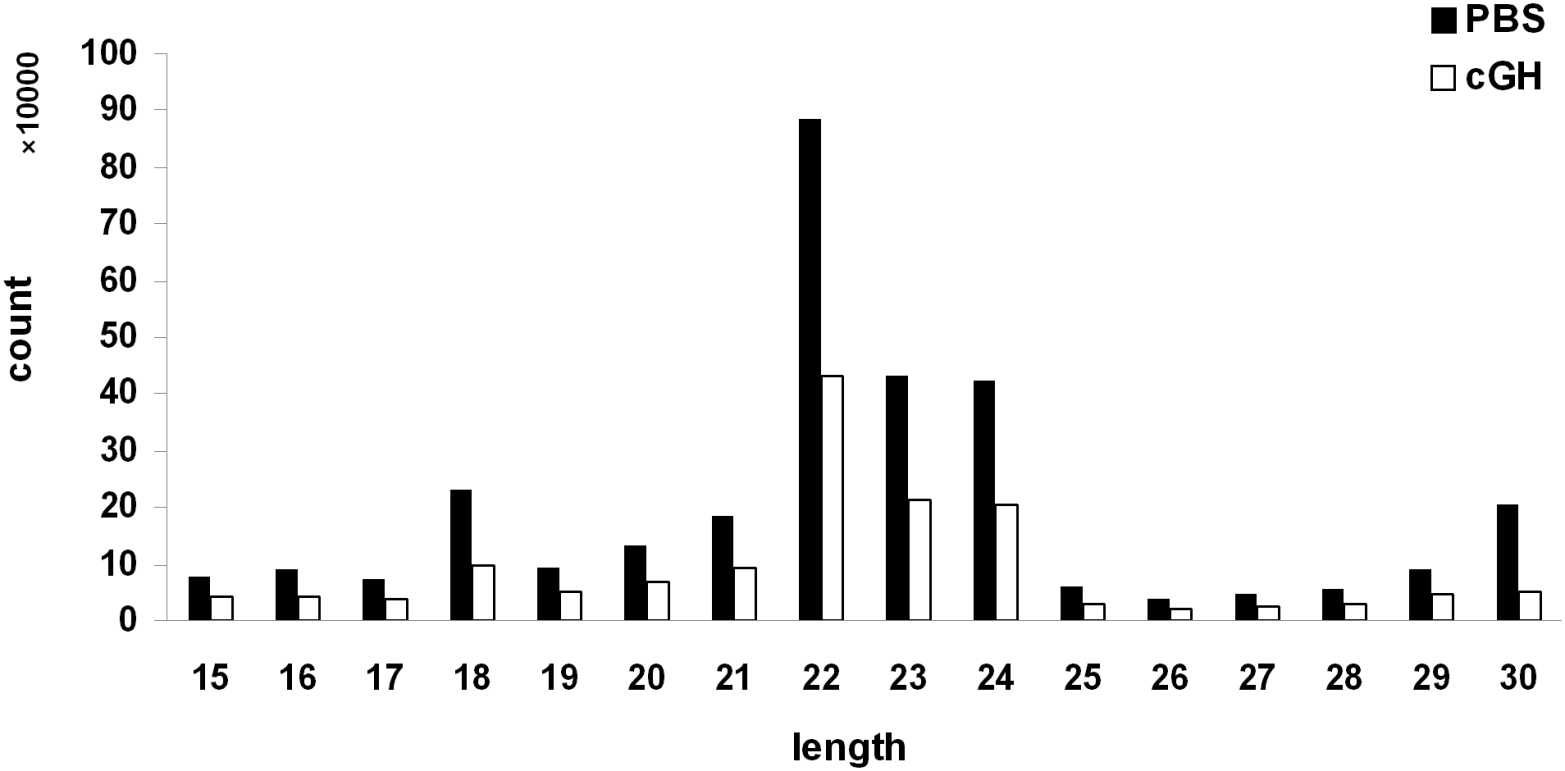
Length distributions of small RNA sequences.

**Figure 3:**
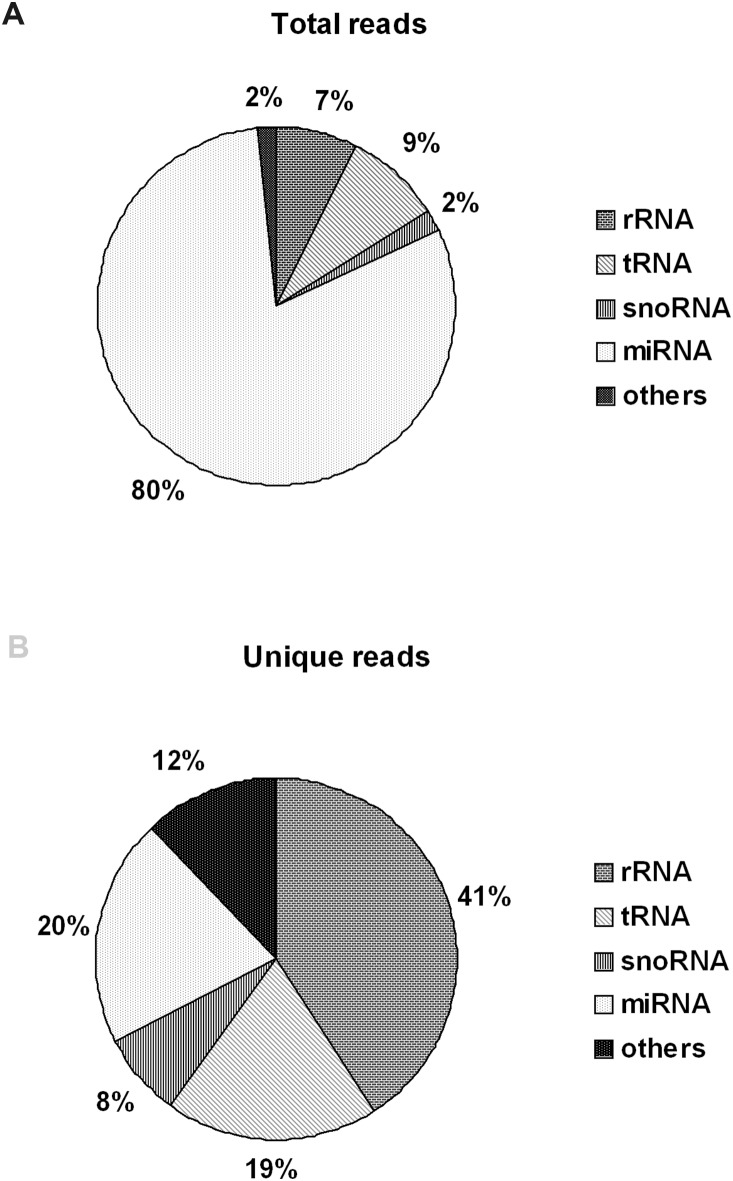
Annotation of small RNA sequences. (A) Annotation of total sequence reads. (B) Annotation of unique sequence reads.

The majority of the 15–30 nt long reads ranged from 18 to 24 nt, and reads of 22 nt long were more abundant than reads of other lengths, indicating that the distribution of the small RNA sequences was consistent with the length range of miRNAs (Figure 2). In other words, most of the 15–30 nt long reads were likely miRNAs. A further analysis showed that most of the small RNAs could be mapped to the chicken genome. After the 15–30 nt reads were counted and grouped to generate unique reads, total reads or unique reads were compared to the sequences in Rfam database and classified. About 75% of total reads matched the sequences in Rfam. As shown in Figure 3, among the matched reads, ∼80% of total reads represented miRNAs, and ∼20% of unique reads represented miRNAs, indicating that the sequenced small RNA reads were enriched with miRNAs.

The unique reads that matched miRNAs in Rfam were BLAST searched against chicken miRNAs in miRBase (20). Of 996 known chicken miRNAs in miRBase, 225 were identified in the present study. Among them, 219 known chicken miRNAs were identified from PBS-treated and 206 were identified from chGH-treated hepatocytes, which corresponded to 491 and 422 known chicken miRNA precursors, respectively (Tables 7 and S4). The remaining reads that did not match known chicken miRNAs were BLAST searched against all the miRNAs in other species, and 264 homologous miRNAs were identified. Of them, 259 were from PBS-treated cells and 234 were from chGH-treated cells (Table S5). The small RNA reads that had no matches in Rfam were analyzed to predict novel miRNAs and their precursors. A total of 95 sequences were predicted to be novel miRNAs, of which 93 were sequenced from PBS-treated cells and 73 from chGH-treated cells (Table S6). We estimated the potential of the precursor sequences for these miRNAs to form stable stem-loop hairpin structures (Figure S1). Interestingly, some of these sequences are related to known chicken miRNAs. For example, the predicted novel chicken miRNA gga-m0015-3p is complementary to the known chicken miRNA gga-miR-126-5p, the 3′ portion of gga-m0016-5p overlaps with the 5′ portion of gga-miR-219b, and the 5′ portion of gga-m0028-3p overlaps with the 3′ portion of gga-miR-3525 (Figure S2).

**Table 7:** Identification of known chicken miRNAs from chicken hepatocytes.

	miRNA	precursor	miRNA (unique)	miRNA (total)	precursor (unique)	precursor (total)
miRBase	996	734	-	-	-	-
PBS	219	491	5,010	1,552,789	8,447	1,958,885
chGH	206	422	4,240	751,527	6,927	948,397

### 4. GH-regulated miRNAs

We estimated the expression levels of miRNAs based on their read numbers. The 10 most abundantly expressed miRNAs in chicken hepatocytes were listed in Table S7. Among the identified known chicken miRNAs, 17 were identified as DEM between PBS-treated and chGH-treated hepatocytes, of which 16 were up-regulated and 1 was down-regulated by chGH. Among the identified chicken miRNAs homologous to miRNAs in other species, 21 were DEM between PBS-treated and chGH-treated hepatocytes, of which 15 were up-regulated and 6 were down-regulated in chGH-treated cells. Among the predicted novel chicken miRNAs, 7 were DEM between PBS-treated and chGH-treated cells, of which 5 were up-regulated and 2 were down-regulated by chGH (Table 8).

**Table 8:** chGH-regulated miRNAs among the identified known and predicted chicken miRNAs.

miRNA	RPM (PBS)	RPM (chGH)	fold change (RPM (chGH/PBS))	p-value
**DEM among the identified known chicken miRNAs**				
up-regulated miRNA				
gga-miR-15b	35.3	63.9	1.8	2.27E-07
gga-miR-19b	263.9	451.0	1.7	5.49E-36
gga-miR-29b	31.5	49.0	1.6	4.71E-04
gga-miR-99a-5p	9.2	15.4	1.7	0.022
gga-miR-146b-3p	3.8	7.5	2.0	0.043
gga-miR-181a-3p	45.0	68.6	1.5	7.91E-05
gga-miR-190	96.0	162.8	1.7	1.02E-13
gga-miR-193a	17.9	30.8	1.7	8.78E-04
gga-miR-194	15.6	23.8	1.5	0.019
gga-miR-223	55.7	94.2	1.7	1.71E-08
gga-miR-455-3p	15.5	24.7	1.6	0.008
gga-miR-1306	0.6	3.7	6.5	0.003
gga-miR-1618-5p	1.1	4.2	3.7	0.011
gga-miR-1628	0.4	2.3	6.1	0.019
gga-miR-1699	1.5	5.6	3.7	0.003
gga-miR-1731	35.9	56.0	1.6	1.72E-04
down-regulated miRNA				
gga-miR-1724	2.3	0.01	0.004	0.023
**DEM among the miRNAs homologous to miRNAs in other species**				
up-regulated miRNA				
aca-miR-16b-5p	3.2	7.9	2.4	0.009
aca-miR-30c-3p	11.1	19.6	1.8	0.005
ahy-miR-3512	21.4	52.7	2.5	2.30E-11
bta-miR-139	11.8	22.4	1.9	0.001
ccr-miR-92a	21.2	43.8	2.1	3.20E-07
ccr-miR-99	1.1	3.7	3.3	0.024
hsa-miR-34a-3p	1.7	4.7	2.7	0.027
hsa-miR-4792	852.8	1356.3	1.6	3.19E-81
mmu-miR-6238	0.8	2.8	3.7	0.036
oan-miR-1335	41.0	71.8	1.8	1.84E-07
ola-miR-122	19.3	30.3	1.6	0.005
ppt-miR-894	9.5	15.9	1.7	0.022
tca-miR-3885-5p	14.3	27.1	1.9	3.19E-04
xtr-miR-210	11.8	24.3	2.0	1.60E-04
xtr-miR-212	1.7	5.1	3.0	0.013
down-regulated miRNA				
ccr-miR-22b	12.2	5.1	0.4	0.005
ccr-miR-26a	75.8	29.4	0.4	9.45E-15
ccr-miR-130c	10.9	4.7	0.4	0.009
cgr-miR-425-5p	13.2	4.7	0.4	7.79E-04
ggo-let-7f	71.2	37.8	0.5	4.46E-08
ggo-miR-146a	34.34	11.2	0.3	4.96E-09
**DEM among the predicted novel miRNAs**				
up-regulated miRNA				
gga-m0060-3p	0.01	1.9	186.6	0.004
gga-m0006-5p	0.01	1.4	139.9	0.014
gga-m0072-3p	0.8	2.8	3.7	0.036
gga-m0085-3p	5.0	12.6	2.5	7.13E-04
gga-m0073-3p	6.9	14.0	2.0	0.004
down-regulated miRNA				
gga-m0018-5p	1.9	0.01	0.005	0.046
gga-m0011-5p	2.9	0.01	0.003	0.008

### 5. Functional analysis of GH-regulated miRNAs

**Figure 4:**
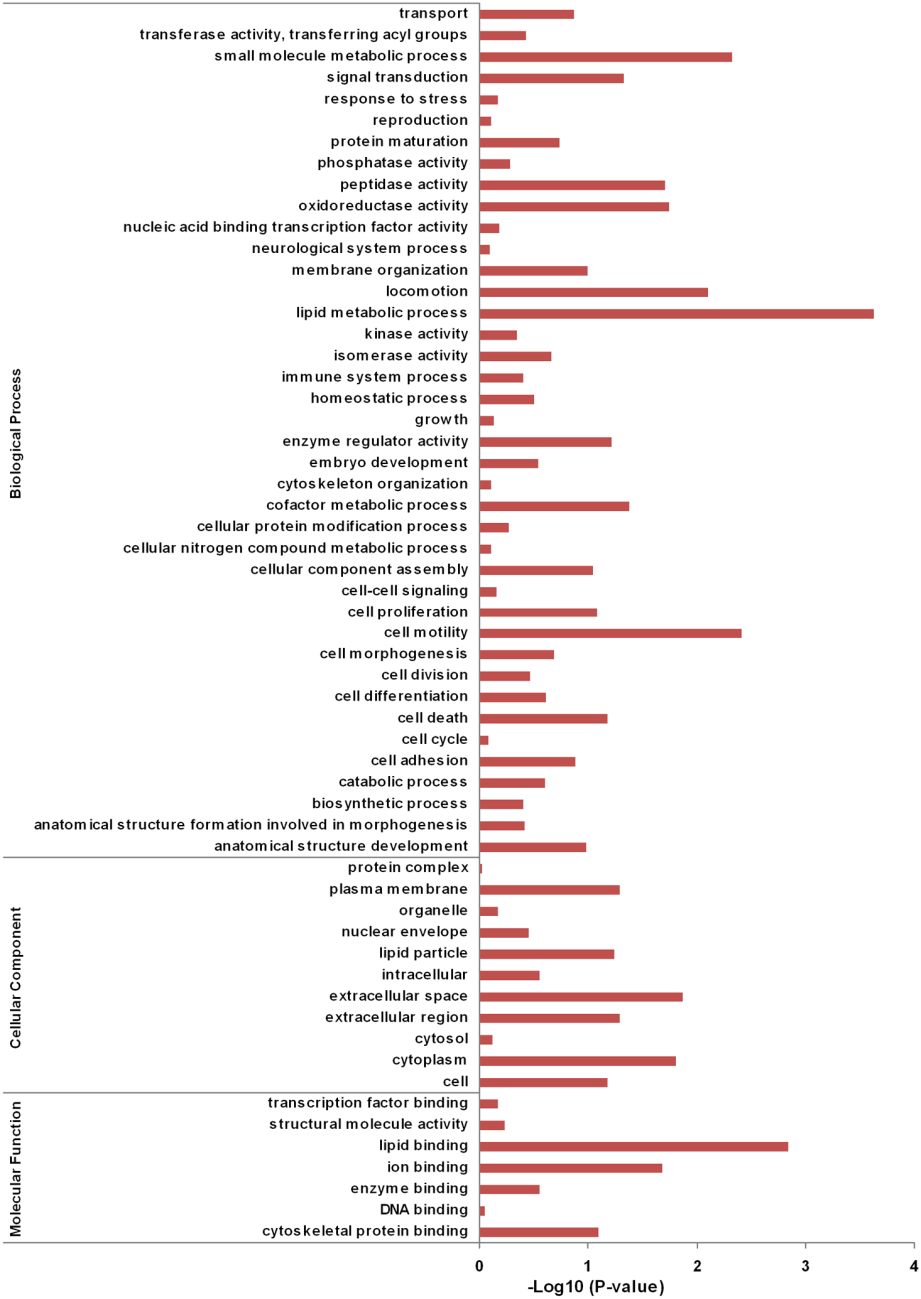
GO enrichment analysis of chGH-regulated mRNAs predicted to be targeted by GH-regulated miRNAs. The target mRNAs for GH up-regulated miRNAs were predicted in the GH down-regulated mRNAs; the target mRNAs for GH down-regulated miRNAs were predicted in the GH up-regulated mRNAs.

The major function of miRNAs is to down-regulate the expression of target mRNAs. To investigate the function of GH-regulated miRNAs, we determined which of the GH-regulated mRNAs could be targeted by the GH-regulated miRNAs. Among the GH down-regulated mRNAs, 32 were predicted as targets of GH up-regulated miRNAs. Among the GH up-regulated mRNAs, 12 were predicted as target genes of GH down-regulated miRNAs (Table 9). A GO enrichment analysis showed that these miRNA target genes were enriched in lipid metabolism, lipid binding, cell motility, and small molecule metabolic process (Figure 4). The most significant GO term was the lipid metabolic process. As shown in Table 10, the GH up-regulated miRNAs targeted 7 genes related to lipid metabolism and the GH down-regulated miRNAs were predicted to target one gene related to lipid metabolism. Among these GH-regulated miRNAs, miR-15b had more predicted target genes related to lipid metabolism than had any other miRs. Several GH-regulated miRNAs were predicted to target the same genes (Table 10).

**Table 9:** GH-regulated chicken genes predicted to be targeted by GH-regulated chicken miRNAs.

miRNA	Target gene
gga-miR-15b	ABCG5, ABHD2, ACSL3, CBLB, ETNPPL, FAM3C, HEPHL1, HIVEP2, KIAA0408, KIAA1107, LIPG, MAST4, MYRIP, NCAM1, PANK1, PLCD1, PPAP2B, RGS6, STAR
gga-miR-19b	ABHD2, AKAP13, ALDH1A3, CBLB, LIPG, MYRIP, PPAP2B, SERPINB6
gga-miR-29b	ABCG5, ABCG8, AKAP13, LIPG, MAST4, PPAP2B, PRRG1, ROBO1, TSKU
gga-miR-146b-3p	ABHD2, ANXA13, PLCD1
gga-miR-190	CBLB, ELOVL6, MYRIP
gga-miR-193a	ABHD2, KIAA0408, MYRIP, PLCD1, PRRG1
gga-miR-194	ABHD2, AKAP13, CBLB, MAST4, MYRIP, PLCD1
gga-miR-223	ALDH1A3, CBLB, MAST4, PLCD1
gga-miR-455-3p	ABHD2, ELOVL6
gga-miR-1306	ABCG8, ANXA13, AKAP13, CCK1R
gga-miR-1618-5p	HEPHL1, MYRIP, SERPINB1
gga-miR-1628	ABHD2, CBLB, LIPG, PANK1, PLCD1, RGS6, SERPINB1, TPPP
gga-miR-1699	ABHD2, AKAP13, HEPHL1, MAST4, PLCD1, PPAP2B, PRRG1, TPPP
gga-miR-1724	AASS, CAMK1D, COQ4, DNAAF2, ECI1, EGLN3, FURIN, FZD5, PXDC1, RETSAT, TMEM229B, TOB2
gga-miR-1731	ABCG5, CCK1R, KIAA1107, PPAP2B, PRRG1, SPTBN1

**Table 10:** Lipid metabolism-related genes predicted to be targeted by chGH-regulated miRNAs.

miRNA	Predicted target gene
up-regulated miRNA	
gga-miR-15b	ACSL3, LIPG, PLCD1, PPAP2B, STAR
gga-miR-19b	ALDH1A3, LIPG, PPAP2B
gga-miR-29b	LIPG, PPAP2B
gga-miR-146b-3p	PLCD1
gga-miR-190	ELOV6
gga-miR-193a	PLCD1
gga-miR-194	PLCD1
gga-miR-223	ALDH1A3, PLCD1
gga-miR-455-3p	ELOV6
gga-miR-1628	LIPG, PLCD1
gga-miR-1699	PLCD1, PPAP2B
gga-miR-1731	PPAP2B
down-regulated miRNA	
gga-miR-1724	RETSAT

## Discussion

In this research through RNA-seq, we found a total of 164 DEG between chGH-treated and untreated chicken hepatocytes. Functional analyses showed most of the GH-regulated genes are involved in liver metabolism, indicating that GH regulates liver metabolism. Lipid metabolism is different between birds and mammals. In mammals lipogenesis occurs in liver, adipose tissue, and mammary gland, whereas in birds this occurs mostly in liver [37]. Lipid metabolic process was identified as a significant GO term among chGH-regulated genes, indicating that GH might play an important regulatory role in lipid metabolism in chicken hepatocytes. In the study of human adipose tissue, Zhao et al. (2011) found the GH-regulated DEG include those that stimulate triglyceride (TG)/ free fatty acid (FFA) cycle, and they also found a new TG hydrolase gene called patatin-like phospholipase domain containing 3 (*PNPLA3*), which could promote TG hydrolysis [38]. Comparing our DEG with those in the above report, we found there was little overlap, indicating the mechanism of GH regulation of lipid metabolism is different between birds and mammals, or between adipose tissue and liver. The chicken *PNPLA4* identified in our study is homologous to *PNPLA3* in mammals, suggesting this gene may play a role in regulating TG hydrolysis in the chicken liver.

In addition to genes that are involved in animal growth, many of the 164 DEG are involved in biosynthetic process (Table S8). This suggests that GH stimulates body growth in chickens not only by stimulating cell proliferation and hypertrophy but also by stimulating nutrient accumulation.

Recent research indicated that multiple signaling pathways are activated by GH, including the JAK2-STAT pathway, the MAPK-ERK1/2 pathway and the PI3K-AKT pathway [12]. In this study, 31 DEG are transcription factors or signaling molecules. Some of them belong to the pathways known to be activated by GH [35], [36] while others belong to pathways that are not known to be activated by GH. Further study of the DEG showed that some of them, for example CAMK1D, had both DNA binding activity and tyrosine kinase activity. Another interesting GH-regulated transcription factor is BCL6. Studies showed that the expression of *BCL6* was controlled by GH through STAT5, and as a transcription factor BCL6 could mediate downstream gene expression [39], [40]. We found that chicken *BCL6* gene has 11 potential STAT5 binding sites (data not shown), implying it could be regulated by chGH through STAT5.

The KEGG pathway analysis of GH-regulated DEG showed the KO terms were mainly related to metabolism, in particular, lipid metabolism. Two DEG were involved in fat digestion and absorption. One of them is *FABP1*, which is an important lipid-related gene [41] and has been shown to be up-regulated by GH in young chickens [42]. The other gene *PPAP2B* is involved in the glycerolipid metabolism pathway.

Among the identified miRNAs in this study, gga-miR-148a is the most abundant miRNA in chicken hepatocytes. This is consistent with the previous report, in which miR-148a was identified as the most abundant miRNA in porcine livers of different breeds [43]. This result indicated miR-148a might play important roles in liver. From many previous reports, miR-122 was known as the most abundant miRNA in liver. But in our study gga-miR-122-5p (previously named gga-miR-122) was identified as the fifth abundant miRNA in primary chicken hepatocytes, similar to the report by Li et al. which found miR-122 was the fourth abundant miRNA in porcine liver [43].

The differentially expressed miRNAs identified in this study might play various important roles in chGH regulation of growth, development and metabolism. miR-223 is expressed higher in the liver of Large White pig (lean type) than in the liver of Tongcheng pig (fatty type) [44]. In this study, we found gga-miR-223 was up-regulated by chGH, indicating it might play a role in lipid metabolism in chicken liver. miR-193a and -190 are expressed at higher levels in the liver of Erhualian pig than in the liver of Large White pig (The two breeds have different rate of lipid metabolism) [43]. In this study gga-miR-193a and 190 were also up-regulated by chGH, indicating they may play roles in lipid metabolism in chicken liver too. miR-15b was another up-regulated miRNA by the chGH treatment. This miRNA in pigs is expressed at a lower level in the liver of Erhualian pig than in the liver of Large White pig, opposite to miR-193a and -190 [43], implying it may play different roles between chickens and pigs. In male rat livers, miR-451 and miR-29b are down-regulated by GH [45]. gga-miR-451 was too down-regulated by GH in chicken hepatocytes in our study, indicating gga-miR-451 might mediate GH regulation of chicken liver metabolism but gga-miR-29b played different roles in GH regulation of liver metabolism comparing to miR-29b in rat. The expression of miR-122 is not changed by GH in male rat liver [45]. We found the same for gga-miR-122 in this study, indicating this miRNA might not be involved in GH regulation of liver metabolism. In this study we also found that some of GH-regulated DEG were potential targets of GH-regulated DEM. GO enrichment analysis showed that these DEG were enriched with genes involved in lipid metabolism, indicating that GH may regulate lipid metabolism through miRNAs. Among the GH-regulated DEM, miR-15b was predicted to target more GH-regulated DEG related to lipid metabolism than any other DEM, including ACSL3, LIPG, PLCD1, PPAP2B and STAR. miR-15b expression was increased in NAFLD models and it lead to inducing the storage of intracellular triglyceride [46]. When ACSL3 increases, triglycerides and free fatty acids reduce [47]. Knock down of ACSL3 decrease hepatic lipogenesis [48]. LIPG has substantial phospholipase activity, and it can reduce plasma concentrations of HDL cholesterol [49]. PLCD1 can catalyze the hydrolysis of membrane lipid phosphatidylinositol 4,5-bisphosphate into second messengers inositol 1,4,5-trisphosphate and diacylglycerol [50]. PPAP2B bioactive lysophospholipids, including lysophosphatidic acid and sphingosine-1-phosphate, and thereby terminates their signaling effects [51]. STAR plays a critical role in the rapid translocation of cholesterol across the outer and inner mitochondrial membranes [52]. It also plays a key role in steroidogenesis by enhancing the metabolism of cholesterol into pregnenolone [53]. This finding indicates miR-15b might be a very important miRNA that mediates the effect of GH on lipid metabolism in chicken liver.

In mammals, the secretion of GH is characterized by an ultradian rhythm and sexual dimorphism. In the male rat, the major bursts of GH secretion occur at regular 3.3 h intervals separated by intervening trough periods with very low or undetectable basal GH levels [54], [55]. GH is also secreted in a pulsatile manner in chickens [56], [57]. In this study, hepatocytes from chickens were treated with one concentration of GH in culture. Therefore, a limitation of the study is that the observed GH-regulated mRNA and miRNA expression profiles may not represent those that occur in the chicken liver *in vivo*.

## Conclusions

This study reveals that GH regulates the expression of many mRNAs involved in metabolism in female chicken hepatocytes, which suggests that GH plays an important role in regulating hepatic metabolism besides somatic growth. This study suggests that GH regulates lipid metabolism in female chicken liver in part by regulating the expression of miRNAs that target the mRNAs involved in lipid metabolism.

## Supporting Information

Figure S1 **Structures of predicted novel miRNAs.** For each novel miRNA, the precursor name, position, strand, length and energy are shown in the first line. The sequence of precursor and total reads are described in the second line. The brackets in the third line denote the secondary structure. The sequence of major unique reads, novel miRNA name and the number of reads are shown below with ‘*’. Then the sequence of each unique reads and the number of reads are shown below with ‘−’.
(TXT)

Figure S2 **Relationship between predicted novel miRNAs and known chicken miRNAs.** For each novel miRNA, the name is listed in the first line. The sequence of known precursor and its name are described in the second line. The sequence of known miRNA and its name are shown below with ‘*’. Then the sequence of novel miRNA and its name are shown below with ‘−’. Note: The sequence of gga-m0015-3p is complementary to gga-miR-126-5p.
(TXT)

Table S1 **Primers for real time RT-PCR.**
(DOC)

Table S2 **Differentially expressed genes by chGH treatment.**
(XLS)

Table S3 **KEGG of differentially expressed genes.**
(XLS)

Table S4 **Identified known chicken miRNAs.**
(XLS)

Table S5 **Identified miRNAs homologous to other species.**
(XLS)

Table S6 **Predicted novel miRNAs.**
(XLS)

Table S7 **Ten most abundant miRNAs.**
(DOC)

Table S8 **Differentially expressed genes related with biosynthetic process.**
(XLS)

## References

[1] Davidson MB (1987) Effect of growth hormone on carbohydrate and lipid metabolism. Endocr Rev 8: 115–131. 3301316 10.1210/edrv-8-2-115

[2] Press M (1988) Growth hormone and metabolism. Diabetes Metab Rev 4: 391–414. 3292176 10.1002/dmr.5610040406

[3] Vijayakumar A, Novosyadlyy R, Wu Y, Yakar S, LeRoith D (2010) Biological effects of growth hormone on carbohydrate and lipid metabolism. Growth Horm IGF Res 20: 1–7. 19800274 10.1016/j.ghir.2009.09.002 PMC2815161

[4] Waters MJ, Brooks AJ (2012) Growth hormone and cell growth. Endocr Dev 23: 86–95. 23182823 10.1159/000341761

[5] Bartke A (2000) Effects of growth hormone on male reproductive functions. J Androl 21: 181–188. 10714810

[6] Hull KL, Harvey S (2001) Growth hormone: roles in female reproduction. J Endocrinol 168: 1–23. 11139766 10.1677/joe.0.1680001

[7] Moller N, Jorgensen JO (2009) Effects of growth hormone on glucose, lipid, and protein metabolism in human subjects. Endocr Rev 30: 152–177. 19240267 10.1210/er.2008-0027

[8] Wells JA (1996) Binding in the growth hormone receptor complex. Proc Natl Acad Sci U S A 93: 1–6. 8552582 10.1073/pnas.93.1.1 PMC40168

[9] Gent J, van Kerkhof P, Roza M, Bu G, Strous GJ (2002) Ligand-independent growth hormone receptor dimerization occurs in the endoplasmic reticulum and is required for ubiquitin system-dependent endocytosis. Proc Natl Acad Sci U S A 99: 9858–9863. 12105275 10.1073/pnas.152294299 PMC125043

[10] Brown RJ, Adams JJ, Pelekanos RA, Wan Y, McKinstry WJ, et al (2005) Model for growth hormone receptor activation based on subunit rotation within a receptor dimer. Nat Struct Mol Biol 12: 814–821. 16116438 10.1038/nsmb977

[11] Argetsinger LS, Campbell GS, Yang X, Witthuhn BA, Silvennoinen O, et al (1993) Identification of JAK2 as a growth hormone receptor-associated tyrosine kinase. Cell 74: 237–244. 8343952 10.1016/0092-8674(93)90415-m

[12] Lanning NJ, Carter-Su C (2006) Recent advances in growth hormone signaling. Rev Endocr Metab Disord 7: 225–235. 17308965 10.1007/s11154-007-9025-5

[13] O’Hea EK, Leveille GA (1968) Lipogenesis in isolated adipose tissue of the domestic chick (Gallus domesticus). Comp Biochem Physiol 26: 111–120. 5758294 10.1016/0010-406x(68)90317-4

[14] Leveille GA, O’Hea EK, Chakbabarty K (1968) In vivo lipogenesis in the domestic chicken. Proc Soc Exp Biol Med 128: 398–401. 5663244 10.3181/00379727-128-33022

[15] Bartel DP (2004) MicroRNAs: genomics, biogenesis, mechanism, and function. Cell 116: 281–297. 14744438 10.1016/s0092-8674(04)00045-5

[16] Chang J, Nicolas E, Marks D, Sander C, Lerro A, et al (2004) miR-122, a mammalian liver-specific microRNA, is processed from hcr mRNA and may downregulate the high affinity cationic amino acid transporter CAT-1. RNA Biol 1: 106–113. 17179747 10.4161/rna.1.2.1066

[17] Krutzfeldt J, Rajewsky N, Braich R, Rajeev KG, Tuschl T, et al (2005) Silencing of microRNAs in vivo with ‘antagomirs’. Nature 438: 685–689. 16258535 10.1038/nature04303

[18] Esau C, Davis S, Murray SF, Yu XX, Pandey SK, et al (2006) miR-122 regulation of lipid metabolism revealed by in vivo antisense targeting. Cell Metab 3: 87–98. 16459310 10.1016/j.cmet.2006.01.005

[19] Rayner KJ, Suarez Y, Davalos A, Parathath S, Fitzgerald ML, et al (2010) MiR-33 contributes to the regulation of cholesterol homeostasis. Science 328: 1570–1573. 20466885 10.1126/science.1189862 PMC3114628

[20] Gerin I, Clerbaux LA, Haumont O, Lanthier N, Das AK, et al (2010) Expression of miR-33 from an SREBP2 intron inhibits cholesterol export and fatty acid oxidation. J Biol Chem 285: 33652–33661. 20732877 10.1074/jbc.M110.152090 PMC2962463

[21] Mortazavi A, Williams BA, McCue K, Schaeffer L, Wold B (2008) Mapping and quantifying mammalian transcriptomes by RNA-Seq. Nat Methods 5: 621–628. 18516045 10.1038/nmeth.1226 PMC13303166

[22] Cloonan N, Forrest AR, Kolle G, Gardiner BB, Faulkner GJ, et al (2008) Stem cell transcriptome profiling via massive-scale mRNA sequencing. Nat Methods 5: 613–619. 18516046 10.1038/nmeth.1223

[23] Trapnell C, Roberts A, Goff L, Pertea G, Kim D, et al (2012) Differential gene and transcript expression analysis of RNA-seq experiments with TopHat and Cufflinks. Nat Protoc 7: 562–578. 22383036 10.1038/nprot.2012.016 PMC3334321

[24] Creighton CJ, Reid JG, Gunaratne PH (2009) Expression profiling of microRNAs by deep sequencing. Brief Bioinform 10: 490–497. 19332473 10.1093/bib/bbp019 PMC2733187

[25] Brooks AJ, Waters MJ (2010) The growth hormone receptor: mechanism of activation and clinical implications. Nat Rev Endocrinol 6: 515–525. 20664532 10.1038/nrendo.2010.123

[26] Ono M, Chia DJ, Merino-Martinez R, Flores-Morales A, Unterman TG, et al (2007) Signal transducer and activator of transcription (Stat) 5b-mediated inhibition of insulin-like growth factor binding protein-1 gene transcription: a mechanism for repression of gene expression by growth hormone. Mol Endocrinol 21: 1443–1457. 17426286 10.1210/me.2006-0543

[27] Campbell RM, Scanes CG (1988) Pharmacological investigations on the lipolytic and antilipolytic effects of growth hormone (GH) in chicken adipose tissue in vitro: evidence for involvement of calcium and polyamines. Proc Soc Exp Biol Med 188: 177–184. 2453890 10.3181/00379727-188-42725

[28] Cupo MA, Cartwright AL (1989) Lipid synthesis and lipoprotein secretion by chick liver cells in culture: influence of growth hormone and insulin-like growth factor-I. Comp Biochem Physiol B 94: 355–360. 2591195 10.1016/0305-0491(89)90355-6

[29] Harvey S (2013) Growth hormone and growth? Gen Comp Endocrinol 190: 3–9. 23376467 10.1016/j.ygcen.2013.01.008

[30] Wang XG, Shao F, Wang HJ, Yang L, Yu JF, et al (2013) MicroRNA-126 expression is decreased in cultured primary chicken hepatocytes and targets the sprouty-related EVH1 domain containing 1 mRNA. Poult Sci 92: 1888–1896. 23776277 10.3382/ps.2012-02919

[31] Ambros V, Bartel B, Bartel DP, Burge CB, Carrington JC, et al (2003) A uniform system for microRNA annotation. RNA 9: 277–279. 12592000 10.1261/rna.2183803 PMC1370393

[32] Chen C, Deng B, Qiao M, Zheng R, Chai J, et al (2012) Solexa sequencing identification of conserved and novel microRNAs in backfat of Large White and Chinese Meishan pigs. PLoS One 7: e31426. 22355364 10.1371/journal.pone.0031426 PMC3280305

[33] Albagli O, Lantoine D, Quief S, Quignon F, Englert C, et al (1999) Overexpressed BCL6 (LAZ3) oncoprotein triggers apoptosis, delays S phase progression and associates with replication foci. Oncogene 18: 5063–5075. 10490843 10.1038/sj.onc.1202892

[34] Marlow R, Strickland P, Lee JS, Wu X, Pebenito M, et al (2008) SLITs suppress tumor growth in vivo by silencing Sdf1/Cxcr4 within breast epithelium. Cancer Res 68: 7819–7827. 18829537 10.1158/0008-5472.CAN-08-1357 PMC3075571

[35] Herrington J, Smit LS, Schwartz J, Carter-Su C (2000) The role of STAT proteins in growth hormone signaling. Oncogene 19: 2585–2597. 10851057 10.1038/sj.onc.1203526

[36] Winston LA, Hunter T (1995) JAK2, Ras, and Raf are required for activation of extracellular signal-regulated kinase/mitogen-activated protein kinase by growth hormone. J Biol Chem 270: 30837–30840. 8537333 10.1074/jbc.270.52.30837

[37] Bergen WG, Mersmann HJ (2005) Comparative aspects of lipid metabolism: impact on contemporary research and use of animal models. J Nutr 135: 2499–2502. 16251600 10.1093/jn/135.11.2499

[38] Zhao JT, Cowley MJ, Lee P, Birzniece V, Kaplan W, et al (2011) Identification of novel GH-regulated pathway of lipid metabolism in adipose tissue: a gene expression study in hypopituitary men. J Clin Endocrinol Metab 96: E1188–1196. 21565791 10.1210/jc.2010-2679

[39] Chen Y, Lin G, Huo JS, Barney D, Wang Z, et al (2009) Computational and functional analysis of growth hormone (GH)-regulated genes identifies the transcriptional repressor B-cell lymphoma 6 (Bc16) as a participant in GH-regulated transcription. Endocrinology 150: 3645–3654. 19406940 10.1210/en.2009-0212 PMC2717871

[40] Zhang Y, Laz EV, Waxman DJ (2012) Dynamic, sex-differential STAT5 and BCL6 binding to sex-biased, growth hormone-regulated genes in adult mouse liver. Mol Cell Biol 32: 880–896. 22158971 10.1128/MCB.06312-11 PMC3272977

[41] Musso G, Gambino R, Cassader M (2009) Recent insights into hepatic lipid metabolism in non-alcoholic fatty liver disease (NAFLD). Prog Lipid Res 48: 1–26. 18824034 10.1016/j.plipres.2008.08.001

[42] Wang X, Carre W, Saxton AM, Cogburn LA (2007) Manipulation of thyroid status and/or GH injection alters hepatic gene expression in the juvenile chicken. Cytogenet Genome Res 117: 174–188. 17675858 10.1159/000103178

[43] Li R, Sun Q, Jia Y, Cong R, Ni Y, et al (2012) Coordinated miRNA/mRNA expression profiles for understanding breed-specific metabolic characters of liver between Erhualian and large white pigs. PLoS One 7: e38716. 22719927 10.1371/journal.pone.0038716 PMC3373568

[44] Xie SS, Li XY, Liu T, Cao JH, Zhong Q, et al (2011) Discovery of porcine microRNAs in multiple tissues by a Solexa deep sequencing approach. PLoS One 6: e16235. 21283541 10.1371/journal.pone.0016235 PMC3026822

[45] Cheung L, Gustavsson C, Norstedt G, Tollet-Egnell P (2009) Sex-different and growth hormone-regulated expression of microRNA in rat liver. BMC Mol Biol 10: 13. 19236699 10.1186/1471-2199-10-13 PMC2654566

[46] Zhang Y, Cheng X, Lu Z, Wang J, Chen H, et al (2013) Upregulation of miR-15b in NAFLD models and in the serum of patients with fatty liver disease. Diabetes Res Clin Pract 99: 327–334. 23287814 10.1016/j.diabres.2012.11.025

[47] Wu M, Cao A, Dong B, Liu J (2011) Reduction of serum free fatty acids and triglycerides by liver-targeted expression of long chain acyl-CoA synthetase 3. Int J Mol Med 27: 655–662. 21347510 10.3892/ijmm.2011.624

[48] Bu SY, Mashek MT, Mashek DG (2009) Suppression of long chain acyl-CoA synthetase 3 decreases hepatic de novo fatty acid synthesis through decreased transcriptional activity. J Biol Chem 284: 30474–30483. 19737935 10.1074/jbc.M109.036665 PMC2781602

[49] Jaye M, Lynch KJ, Krawiec J, Marchadier D, Maugeais C, et al (1999) A novel endothelial-derived lipase that modulates HDL metabolism. Nat Genet 21: 424–428. 10192396 10.1038/7766

[50] Sidhu RS, Clough RR, Bhullar RP (2005) Regulation of phospholipase C-delta1 through direct interactions with the small GTPase Ral and calmodulin. J Biol Chem 280: 21933–21941. 15817490 10.1074/jbc.M412966200

[51] Ren H, Panchatcharam M, Mueller P, Escalante-Alcalde D, Morris AJ, et al (2013) Lipid phosphate phosphatase (LPP3) and vascular development. Biochim Biophys Acta 1831: 126–132. 22835522 10.1016/j.bbalip.2012.07.012 PMC3683602

[52] Estabrook RW, Rainey WE (1996) Twinkle, twinkle little StAR, how we wonder what you are. Proc Natl Acad Sci U S A 93: 13552–13554. 8942971 10.1073/pnas.93.24.13552 PMC33645

[53] Sugawara T, Lin D, Holt JA, Martin KO, Javitt NB, et al (1995) Structure of the human steroidogenic acute regulatory protein (StAR) gene: StAR stimulates mitochondrial cholesterol 27-hydroxylase activity. Biochemistry 34: 12506–12512. 7547998 10.1021/bi00039a004

[54] Tannenbaum GS, Martin JB (1976) Evidence for an endogenous ultradian rhythm governing growth hormone secretion in the rat. Endocrinology 98: 562–570. 1261487 10.1210/endo-98-3-562

[55] Stroh T, van Schouwenburg MR, Beaudet A, Tannenbaum GS (2009) Subcellular dynamics of somatostatin receptor subtype 1 in the rat arcuate nucleus: receptor localization and synaptic connectivity vary in parallel with the ultradian rhythm of growth hormone secretion. J Neurosci 29: 8198–8205. 19553459 10.1523/JNEUROSCI.0336-09.2009 PMC6666050

[56] Johnson RJ (1988) Diminution of pulsatile growth hormone secretion in the domestic fowl (Gallus domesticus): evidence of sexual dimorphism. J Endocrinol 119: 101–109. 3193042 10.1677/joe.0.1190101

[57] Buonomo FC, Lauterio TJ, Scanes CG (1984) Episodic growth hormone secretion in the domestic fowl (Gallus domesticus): alpha adrenergic regulation. Comp Biochem Physiol C 78: 409–413. 6149089 10.1016/0742-8413(84)90107-5

