# Supplementary material for: Growth Hormone-Regulated mRNAs and miRNAs in Chicken Hepatocytes
Source: PLoS One. 2014 Nov 11;9(11):e112896. doi: 10.1371/journal.pone.0112896 (PMC4227886; doi:10.1371/journal.pone.0112896)
Supplement: Table S1 — Primers for real time RT-PCR. (DOC) [file pone.0112896.s003.doc]

Table S1 Primers for real time RT-PCR

| Gene | Primer (5′ to 3′) | Product size (bp) |
| --- | --- | --- |
| FABP1 | F: GGAGAGAAGGCCAAGTGTAT  R: ATTGTATGGGTGATGGTGTC | 115 |
| FGFR3 | F: CAGAGTGACAGATTCCCCTT  R: TCCTGTGCTCTCCCTTGAAT | 221 |
| FURIN | F: CCAATAACTACGGCACACTG  R: TCCATGCTGTTCTCCAGGTT | 238 |
| IRF8 | F: GCAGCATCATTCGGGCTACT  R: GCTTCTTGGTGATCTGCTTC | 221 |
| LPIN1 | F: CACCCATTCAAAGCCTGTCA  R: TGCCTTTGTAGCCTGTGGTA | 143 |
| MAPKAPK3 | F: TCCTTATTATGTGGCCCCAG  R: GTGGCGAATCAGTTGTTTGG | 235 |
| PHGDH | F: CCGAAAGAAGTACATGGGCA  R: AGGTCTCAGGGGTGATGATG | 146 |
| PKIG | F: TGGAAGGGGCAGAGAGTGAA  R: GTCCTGGTTGGCTTTTGTCC | 189 |
| THRSP | F: GACTCCAAGGGTGATCTGAC  R: TCATTTCTGCCCTGCCTACT | 207 |
| ABCG8 | F: AGCCTGGAACAACTCTGGAC  R: CCCAGAGCCTTGGTTACAAGT | 167 |
| ALDH1A3 | F: GGAAGGAGACAAGCCTGATG  R: TCCCGTGTCCATTGTTTCTA | 178 |
| BCL6 | F: CACAGCGACAAACCCTACAA  R: TGGGCAACCTGGACAAATCT | 227 |
| LIPG | F: TGCCATTCAAAGTCTACCAC  R: CAAAGAGAGGGGTTCGGAGT | 128 |
| NECAB1 | F: CTCGGCAGGAAAGACAAGGT  R: TTGAGGGTTAGTGGGCGATA | 115 |
| PDE10A | F: GAAGTCAGCAGGTATCAGGA  R: GCCGTGGTTGTCCTTCTTTA | 232 |
| PPAP2B | F: TGGCTTACTGCGTGGTGTTT  R: TGATGAGATTTGAGAGCAGC | 237 |
| RGS6 | F: GAGGATCAAGGGGAAGCAAT  R: ACGGTGCCTGAAAACGATAG | 124 |
| STAR | F: AAGAGGTGAAGATCCTCCA  R: CTGATAAATCCCTGCTGCTC | 198 |
| FGF1 | F: TGTATGGCTCGCAGCTACCA  R: GGCAATGGGAGGAAGAGGAT | 190 |
| GPX7 | F: GGCTCGGTGTCGTTAGTTGT  R: TTGGTGTCTGGTTCTTGCTG | 158 |
| MEF2A | F: ATGCCCACTGCCTACAACAC  R: CCAGAAACGAGAGAGCTGAG | 155 |
| NME1 | F: ATGAAGCTGACACACGCCTC  R: AAGACCCTCCCACACCATAG | 132 |
| PDGFB | F: AGAGGCGAAGCCTTGATGCT  R: CACACCACGAAGTTGGCATT | 127 |
| PNPLA4 | F: GACTTAGGGAAGGCATAGAA  R: CCACCATCAACCCACTTCTC | 226 |
| PRKAR2B | F: ATCATTGCTCAGGGCGACAT  R: GCTCGTGGTTTGTTAGTTAC | 185 |
| PTK2B | F: GAGAACCTGAAGCCCAAGCA  R: CAATGTGAGCAAAGGTGGAG | 124 |
| PTPRC | F: ATCAACTGGTGCCTCTCCCA  R: GATGACTGGTGAGGTGGTGT | 193 |
| ROMO1 | F: TCAGGATCGGCATGAGAGGA  R: CAGGGACGCTTTGGATTAGA | 148 |
| ACER2 | F: AGTTGTGGTCGGAATTGGAT  R: TAATGAAGGCAAGGCAGGTA | 215 |
| ADPGK | F: GGAGTATCAAGCAGGTGAAG  R: AGTCCTGAAAGCACCACAAG | 159 |
| ATF7 | F: CTGAGCAATGAAGTGACGCT  R: TAGCCCTGGGTCTTCTTCTG | 110 |
| BIRC5 | F: GGAGGAACACAAAAAGCACT  R: TTGGTCCGCTTTTTATCCAG | 118 |
| CDK2AP1 | F: CAGTCAAGTACCGCAAAGCA  R: AATGCCTCGTTTTAGCCTCT | 121 |
| DUSP28 | F: GACGCCTTTGAGGTGGTGAA  R: CAGCAATGTGTGGAGGTGTT | 161 |
| FTO | F: TGGAGCTTATGACGAGCCTG  R: TTCTGACTGGCATCTTGCTG | 149 |
| HGF | F: CTCTGCTCTGAAGTTGAATG  R: TGTTTGTGTGGTCTCTGAAG | 116 |
| MAFK | F: GGCATTAAAGGTAAAGGAGG  R: CTGCTTCAGACGGATGACCT | 136 |
| NT5M | F: TGAGCGAGAAAGCCATCAGC  R: ACCGTCTTATCCCGTGTCAG | 238 |
| RUNX2 | F: CCAAGAAGGCACAGACAGAA  R: TGGCTCAAGTAGGACGGGTA | 248 |
| IGF-I | F: TTAACCAGTTCTGCTGCTGC  R: TGGTGTAAGCGTCTACTGCT | 140 |
| CIS | F: GCTACCTGTTCACACTCTCG  R: CGTGACGTAGTGCTGGATGA | 158 |
| SOCS1 | F: GTTTCAGCCTGGATGGCAG  R: GTCTTCACGATGCTCTTCCG' | 154 |
| SOCS2 | F: GTATCTGCTGACCATCTCGG  R: GTTGAACTGCTTGAGCCTCG | 124 |
| SOCS3 | F: CACGCTACAAGCACAAGAGC  R: TACAGCACCCACAGTCCGA | 200 |
| GAPDH | F: TCTTCACCACCATGGAGAAG  R: CAGGACGCATTGCTGACAAT | 154 |
