# Supplementary material for: Growth Hormone-Regulated mRNAs and miRNAs in Chicken Hepatocytes
Source: PLoS One. 2014 Nov 11;9(11):e112896. doi: 10.1371/journal.pone.0112896 (PMC4227886; doi:10.1371/journal.pone.0112896)
Supplement: Table S7 — Ten most abundant miRNAs. (DOC) [file pone.0112896.s009.doc]

Table S7 10 most abundant miRNAs

| miRNA | PBS reads | chGH reads | RPM (PBS) | RPM (chGH) |
| --- | --- | --- | --- | --- |
| gga-miR-148a | 311131 | 161343 | 59371.88 | 75248.77 |
| gga-miR-146c-5p | 245642 | 120052 | 46874.88 | 55991.06 |
| gga-miR-22-3p | 222179 | 92348 | 42397.53 | 43070.19 |
| gga-miR-21 | 175795 | 80677 | 33546.26 | 37626.95 |
| gga-miR-122-5p | 117942 | 63306 | 22506.4 | 29525.29 |
| gga-miR-26a | 76805 | 34038 | 14656.39 | 15874.99 |
| gga-miR-30d | 61624 | 29856 | 11759.46 | 13924.54 |
| gga-miR-30a-5p | 35146 | 19413 | 6706.771 | 9054.03 |
| gga-miR-92 | 34288 | 15137 | 6543.042 | 7059.746 |
| gga-miR-101 | 29679 | 15991 | 5663.525 | 7458.044 |
